# Sensitivity of diagnostic tests for human soil-transmitted helminth infections: a meta-analysis in the absence of a true gold standard

**DOI:** 10.1016/j.ijpara.2014.05.009

**Published:** 2014-10-01

**Authors:** Birgit Nikolay, Simon J. Brooker, Rachel L. Pullan

**Affiliations:** Faculty of Infectious and Tropical Diseases, London School of Hygiene & Tropical Medicine, Keppel Street, WC1E 7HT London, United Kingdom

**Keywords:** Soil transmitted helminths, Diagnostics, Meta-analysis, Latent class analysis

## Abstract

Reliable, sensitive and practical diagnostic tests are an essential tool in disease control programmes for mapping, impact evaluation and surveillance. To provide a robust global assessment of the relative performance of available diagnostic tools for the detection of soil-transmitted helminths, we conducted a meta-analysis comparing the sensitivities and the quantitative performance of the most commonly used copro-microscopic diagnostic methods for soil-transmitted helminths, namely Kato-Katz, direct microscopy, formol-ether concentration, McMaster, FLOTAC and Mini-FLOTAC. In the absence of a perfect reference standard, we employed a Bayesian latent class analysis to estimate the true, unobserved sensitivity of compared diagnostic tests for each of the soil-transmitted helminth species *Ascaris lumbricoides*, *Trichuris trichiura* and the hookworms. To investigate the influence of varying transmission settings we subsequently stratified the analysis by intensity of infection. Overall, sensitivity estimates varied between the different methods, ranging from 42.8% for direct microscopy to 92.7% for FLOTAC. The widely used double slide Kato-Katz method had a sensitivity of 74–95% for the three soil-transmitted helminth species at high infection intensity, however sensitivity dropped to 53–80% in low intensity settings, being lowest for hookworm and *A. lumbricoides*. The highest sensitivity, overall and in both intensity groups, was observed for the FLOTAC method, whereas the sensitivity of the Mini-FLOTAC method was comparable with the Kato-Katz method. FLOTAC average egg count estimates were significantly lower compared with Kato-Katz, while the compared McMaster counts varied. In conclusion, we demonstrate that the Kato-Katz and Mini-FLOTAC methods had comparable sensitivities. We further show that test sensitivity of the Kato-Katz method is reduced in low transmission settings.

## Highlights

• A Bayesian latent class meta-analysis of diagnostic tests for soil-transmitted helminths was performed.
• Overall sensitivity of evaluated diagnostic tests was low.
• Test performance was strongly influenced by intensity of infection.
• FLOTAC method sensitivity was highest overall and in both intensity groups.
• The performance of the Kato-Katz method in high intensity settings was acceptable.

## Graphical abstract

**Figure:**
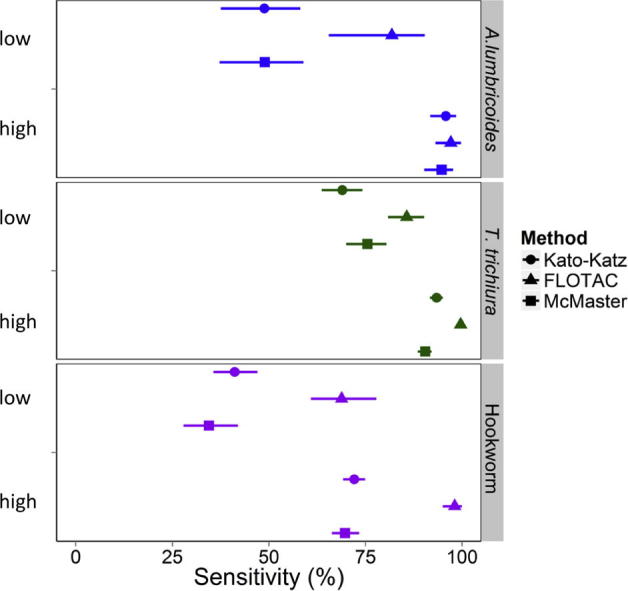

## 1 Introduction

Reliable, sensitive and practical diagnostic tests are an essential tool in disease control programmes, including those for neglected tropical diseases. The requirements and expectations for a diagnostic tool in terms of technical performance, feasibility and costs change as control programmes progress through different phases, from initially high levels of infections to the confirmation of absence of infections. More precisely, during initial mapping to identify priority areas for control, when infection levels are typically highest, a diagnostic test with moderate sensitivity is acceptable, although the chosen tool needs to be easy to use, cost-effective and allow for the high-throughput screening of large populations (McCarthy et al., 2012, Solomon et al., 2012). Since mapping data can also serve as a baseline for the monitoring and evaluation of programme impact, diagnostic tests must have sufficient performance to detect changes in the prevalence and intensity of infection (Solomon et al., 2012). In later stages of programmes, when infection prevalence and intensity have decreased significantly, more sensitive diagnostic tools are needed to establish an endpoint of treatment programmes. If test sensitivity is insufficient at this point, light infections might be missed and this runs the risk of stopping control programmes too early, before programme endpoints have been achieved. Highly sensitive tests are also required for surveillance once treatment has been stopped to detect the potential re-occurrence of infections (McCarthy et al., 2012, Solomon et al., 2012). Finally, diagnostic tests play an important role in the assessment of treatment efficacy (Albonico et al., 2012) and in patient management.

For the detection of the human soil-transmitted helminth (STH) species, *Ascaris lumbricoides*, *Trichuris trichiura* and the hookworms (*Necator americanus* and *Ancylostoma duodenale*), The World Health Organization (WHO) currently recommends the use of the Kato-Katz method, based on duplicate slides (WHO, 2002). Other commonly used methods include direct smear microscopy, formol-ether concentration (FEC), McMaster, FLOTAC and Mini-FLOTAC. All of these techniques rely on visual examination of a small sample of stool to determine the presence and number of STH eggs (WHO, 1994). Due to intra- and inter-sample variation in egg counts (Booth et al., 2003, Krauth et al., 2012), microscopy-based techniques can have differing sensitivities, especially in low transmission settings. Moreover, diagnostic methods vary considerably in the quantification of egg counts, which is necessary to establish intensity of infection and to evaluate treatment effects (Knopp et al., 2011, Albonico et al., 2012, Levecke et al., 2014). In order to better understand the suitability of diagnostic tools for various transmission settings and stages of disease control programmes, we performed a meta-analysis of the most commonly used copro-microscopic STH diagnostic tests.

Our main study objective was an independent and global assessment of the relative performance of commonly used diagnostic methods for STH, as well as factors associated with heterogeneity in test sensitivity. Previous evaluations of STH diagnostics have generally relied on comparisons with a combined reference standard (generated by adding the results of several compared tests or consecutively obtained samples), an approach which has been widely criticised (Enoe et al., 2000, Ihorst et al., 2007). Moreover, the absence of a common reference standard has been a major obstacle for combined evaluations of diagnostic tests in the form of a meta-analysis. We have addressed this problem by using Bayesian latent class analysis (LCA), which allows simultaneous estimation of the unknown true prevalence of infection and the sensitivities and specificities of compared diagnostic tests. This approach has been previously applied to the evaluation of imperfect diagnostic tests for Chagas disease, leishmaniasis and malaria (Menten et al., 2008, de Araujo Pereira et al., 2012, Goncalves et al., 2012), as well as specific studies evaluating STH diagnostic methods (Booth et al., 2003, Tarafder et al., 2010, Assefa et al., 2014, Knopp et al., 2014). The approach has also been used for the meta-analyses of diagnostic test performance (Ochola et al., 2006, Menten et al., 2008, Limmathurotsakul et al., 2012). The current paper presents a Bayesian meta-analysis of different diagnostic tests for the detection of STH species.

## 2 Materials and methods

### 2.1 Literature search

A systematic literature search was performed to identify publications presenting the evaluation of diagnostic techniques for the human STH species, *A. lumbricoides*, *T. trichiura* and hookworms (*N. americanus* and *A. duodenale*). Systematic searches were performed (date of search 25th February 2014) using the electronic databases PubMed (http://www.ncbi.nlm.nih.gov/), MEDLINE and EMBASE (via OvidSP) (http://ovidsp.uk.ovid.com/) and the medical subject headings and search terms as detailed in Supplementary Data S1. Articles were considered if written in English, German, French or Spanish. The search was validated by verifying that a number of previously identified key readings were included in the retrieved search results. The titles of initially obtained search results were screened for suitable content and all abstracts mentioning studies on helminths were retrieved. The abstracts were subsequently screened for studies using more than one diagnostic test for the determination of infections, even if not directly mentioning a comparison of test performances. Full texts were read and information on test outcomes, egg counts, age-groups, countries of the studies and years of publication was extracted where results were presented in a suitable format as explained below. Reference lists were screened for additional publications.

**Fig. 1:**
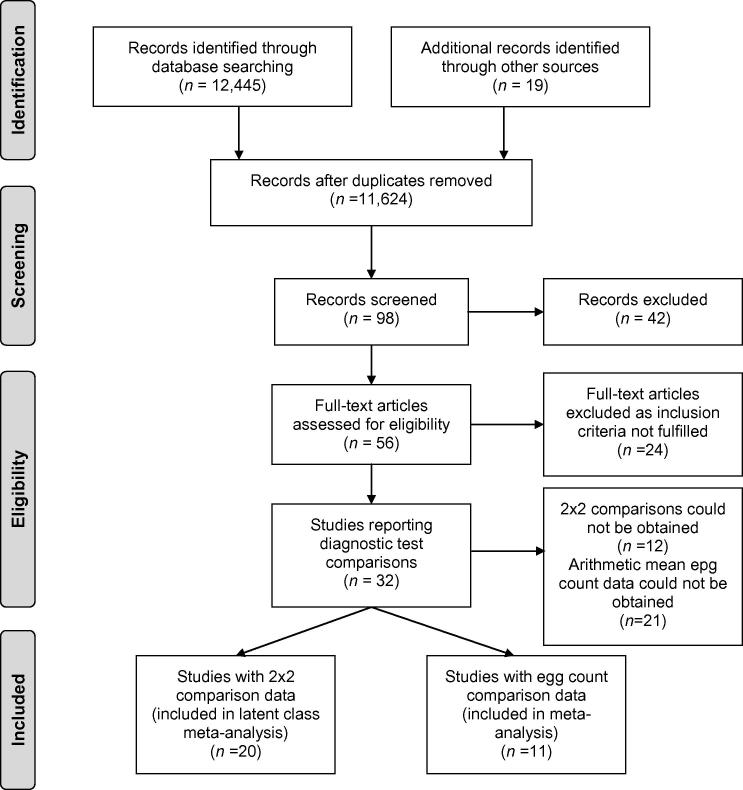
Literature search and selection. Included were studies evaluating selected diagnostic methods (Kato-Katz, direct microscopy, formol-ether concentration, McMaster, FLOTAC, Mini-FLOTAC) in field settings. The results of selected diagnostic test comparisons were presented in 32 articles; 2 × 2 comparisons could be extracted for 20 articles that were finally included in the meta-analysis.

The literature selection process is outlined in Fig. 1. Data were collected separately for *A. lumbricoides*, *T. trichiura* and hookworms, and restricted to the most commonly used diagnostic methods for STH, namely Kato-Katz (Katz et al., 1972), direct microscopy (WHO, 1994), formol-ether concentration (FEC) (Ritchie, 1948), McMaster (Ministry of Agriculture Fisheries and Food, 1986), FLOTAC (Cringoli et al., 2010) and Mini-FLOTAC (Barda et al., 2013a). Other techniques such as midi-Parasep, Koga Agar Plate, Willis technique and Spontaneous tube sedimentation technique (SSTT) were not included due to a lack of suitable data. As performance during field surveys was the main interest, evaluations of diagnostic tests on samples from diagnostic laboratories of hospitals were excluded. Only data provided in the form of 2 × 2 comparisons (T1+T2+, T1+T2−, T1−T2+, T1−T2−, where T1 and T2 are the two diagnostic methods and + and − indicate the observed positive or negative results) were retained. This also included data for which these 2 × 2 comparisons could be created by transforming the original data provided, e.g. where comparisons were made against a combined ‘gold standard’ of two diagnostic methods. Additionally, data on egg counts obtained by the various techniques were retrieved, including those studies that did not provide data in a suitable format for the LCA. Arithmetic mean egg counts were the most commonly reported measures and therefore used for the analysis.

For articles where data could not be directly extracted, corresponding authors were invited to contribute additional study results. Three authors replied and provided four datasets for the analysis; we were also able to contribute a further two datasets to the analysis.

### 2.2 Bayesian LCA

A Bayesian latent class model was used to estimate the sensitivity of different diagnostic tests as described elsewhere (Dendukuri and Joseph, 2001, Branscum et al., 2005). LCA allows estimation of the sensitivity and specificity of imperfect diagnostic tests by assuming a probabilistic model for the relationship between five unobserved, or latent, parameters: true disease prevalence π*_k_* and the sensitivities $S_{i}$, $S_{j}$ and specificities $C_{i}$, $C_{j}$ of diagnostic methods *i* and *j* (Pepe and Janes, 2007). The model additionally incorporates the covariance terms ${\mathrm{covD}}_{\mathrm{ij}}^{+}$, ${\mathrm{covD}}_{\mathrm{ij}}^{-}$ to account for conditional dependency between compared diagnostic tests amongst infected and non-infected individuals, which is necessary as the included diagnostic tests are based on the same biological principle (detection of eggs under a microscope) and therefore factors other than the true infection status are likely to influence both test outcomes simultaneously (Dendukuri and Joseph, 2001). Thus, the joint distribution of the results of a 2 × 2 table follows a multinomial distribution, $\left(X_{k++}\text{,}X_{k+-}\text{,}X_{k-+}\text{,}X_{k--})∼\mathrm{Multi}(p_{k++}\text{,}p_{k+-}\text{,}p_{k-+}\text{,}p_{k--}\text{,}N_{k}\right)$ with the multinomial probabilities calculated as follows:

$$p_{k++}=P(T_{i}^{+}\text{,}T_{j}^{+}|k^{\text{th}}\;\text{population})=[S_{i}S_{j}+{\mathrm{covD}}_{\mathrm{ij}}^{+}]\pi_{k}+[(1-C_{i})(1-C_{j})+{\mathrm{covD}}_{\mathrm{ij}}^{-}](1-\pi_{k})$$

$$p_{k+-}=P(T_{i}^{+}\text{,}T_{j}^{-}|k^{\text{th}}\;\text{population})=[S_{i}(S_{j}-1)-{\mathrm{covD}}_{\mathrm{ij}}^{+}]\pi_{k}+[(1-C_{i})C_{j}-{\mathrm{covD}}_{\mathrm{ij}}^{-}](1-\pi_{k})$$

$$p_{k-+}=P(T_{i}^{-}\text{,}T_{j}^{+}|k^{\text{th}}\;\text{population})=[(S_{i}-1)S_{j}-{\mathrm{covD}}_{\mathrm{ij}}^{+}]\pi_{k}+[C_{i}(1-C_{j})-{\mathrm{covD}}_{\mathrm{ij}}^{-}](1-\pi_{k})$$

$$p_{k--}=P(T_{i}^{-}\text{,}T_{j}^{-}|k^{\text{th}}\;\text{population})=[(S_{i}-1)(S_{j}-1)+{\mathrm{covD}}_{\mathrm{ij}}^{+}]\pi_{k}+[C_{i}C_{j}+{\mathrm{covD}}_{\mathrm{ij}}^{-}](1-\pi_{k})$$

The conditional correlations between two test outcomes for infected and non-infected individuals were calculated as $\rho_{D^{+}}=\frac{{\mathrm{covD}}^{+}}{\sqrt{S_{i}(1-S_{i})S_{j}(1-S_{j})}}$ and $\rho_{D^{-}}=\frac{{\mathrm{covD}}^{-}}{\sqrt{C_{i}(1-C_{i})C_{j}(1-C_{j})}}$, respectively. Uninformative prior information was provided for the sensitivity and underlying true prevalence (using a beta distribution with the shape parameters alpha and beta equal to 1). For the covariance terms, a uniform prior distribution was assumed with limits as described in Dendukuri and Joseph (2001) and Branscum et al. (2005) to ensure that probabilities are confined to values between 0 and 1. Specificity was included as a fixed term based on the most parsimonious, best-fitting model (i.e. that with the lowest deviance information criterion (DIC) value) and was assumed to be the same for all compared methods. This was justified on the dual assumption that false positives are rarely obtained by any type of copro-microscopic technique (Knopp et al., 2011, Levecke et al., 2011) and the necessity to restrict the number of estimated parameters for the identifiability of the model. The models, built separately for *A. lumbricoides*, *T. trichiura* and hookworms, were computed using WinBUGS software version 14 (Spiegelhalter, D., Thomas, A., Best, N., Gilks, W., 1996. BUGS: Bayesian Inference Using Gibbs Sampling. MRC Biostatistics Unit, Cambridge).

Models were also developed separately for low and high intensity settings. Stratification was based on reported arithmetic mean egg counts (in eggs per gram of faeces, epg). Empirical cut-offs of 2500 epg, 400 epg and 165 epg average infection intensity were used for *A. lumbricoides*, *T. trichiura* and hookworms, respectively. These cut-offs were established based on the overall average infection intensity of studies included in the meta-analysis. Data with only geometric means reported were excluded from this analysis unless the geometric mean, which is lower than the average egg count, exceeded the cut-off value.

Further details of model parameterisation, including handling of multiple slides, are provided in Supplementary Data S2.

### 2.3 Comparison of quantitative performances

To compare the various diagnostic tests in terms of their quantitative performance, we compared the arithmetic mean egg count obtained by various techniques. Statistical significance of differences was assessed using the non-parametric paired Wilcoxon signed-ranks test and the linearity of the relationship between counts was assessed by scatter plots of log-transformed (natural logarithm) average egg counts. Moreover, we evaluated the percentage of studies reporting egg counts of other techniques that were lower/higher than the Kato-Katz method, which currently forms the basis of the WHO defined intensity thresholds. To allow for a small variation in counts, egg counts were considered as lower or higher than the Kato-Katz method if these were lower or higher than the Kato-Katz egg count plus or minus 10%. Due to the limited availability of data and the fact that faecal egg counts do not vary significantly by the sampling effort for Kato-Katz analysis, all versions of Kato-Katz were combined (Levecke et al., 2014).

## 3 Results

### 3.1 Identification of diagnostic test comparisons

**Fig. 2:**
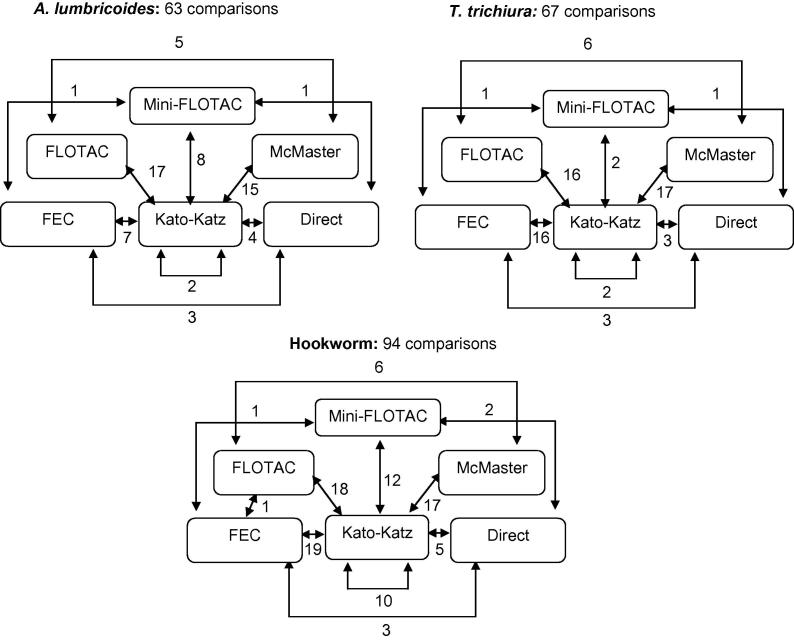
Two-by-two comparisons of diagnostic methods by soil-transmitted helminth species *Acaris lumbricoides, Trichuris trichiuria* and hookworm. The outlined comparisons were included in the models; numbers represent the number of available comparisons. Where studies could be subdivided into several populations, each was counted as one comparison. The Kato-Katz method could be differentiated into variations of the protocol according to number of slides or samples processed.

The initial literature search identified 56 articles which were retrieved for full-text review. Of these, 32 studies fulfilled the inclusion criteria and 2 × 2 comparison data could be obtained for 20 studies (Table 1) (see Fig. 1 for an outline of literature selection steps). The number of extracted 2 × 2 comparisons by species and diagnostic methods is shown in Fig. 2. The included studies were published between 2003 and 2014 and conducted in 12 countries, primarily among school-aged children. The inclusion of only recent studies was somewhat surprising. Even though the original literature search had retrieved studies published since 1967, the non-availability of 2 × 2 data, the type of compared techniques and the evaluation of methods in laboratory or hospital samples led to their exclusion. The evaluation of diagnostic tests was mainly based on comparison with a combined reference-standard (14 of 20 studies); few studies used predicted estimates as a reference (1/20), an LCA approach (1/20) or a combination of the two (1/20). Three studies did not provide sensitivity estimates. The most widely applied method was the Kato-Katz method in 18 of 20 studies (mostly 1-slide or 2-slides on a single sample). The main characteristics of included studies are summarised in Table 1.

**Table 1:** Studies included in a soil-transmitted helminth (STH) diagnostic test meta-analysis. The literature search identified 20 studies evaluating selected diagnostic methods in field settings for which 2 × 2 test comparisons could be obtained. The majority of studies compared diagnostic test performance with a combined reference standard obtained by adding positive test results from all evaluated methods. One additional study (Funk et al., 2013) was included for the evaluation of quantitative test performances. Analysis 1 refers to studies used for the latent class analysis of test sensitivities, while analysis 2 indicates studies used for the comparison of egg count outcomes.

Reference	Country	Age group	Sample size	Compared methods	Reference standard	STH species	Analysis
Albonico et al. (2012)	Tanzania (Zanzibar)	SAC	430	Kato-Katz, McMaster	Combined	*Ascaris lumbricoides, Trichuris trichiura*, hookworm	1, 2
Albonico et al. (2013)^b^	Tanzania (Zanzibar)	SAC	304, 231^a^	Kato-Katz, McMaster, FLOTAC	Combined	*A. lumbricoides, T. trichiura*, hookworm	1, 2
Arias and Urrego (2013)	Colombia	all	309	Direct, FEC	Combined	*A. lumbricoides, T. trichiura, hookworm*	1
Assefa et al. (2014)	Kenya	SAC	132^a^	Kato-Katz, Mini-FLOTAC	LCA	*A. lumbricoides, T. trichiura*, hookworm	1, 2
Barda et al. (2013a)^b^	Tanzania, India	SAC	100, 80	Direct, FEC, Mini-FLOTAC	Combined	*A. lumbricoides, T. trichiura*, hookworm	1
Barda et al. (2013b)^b^	Tanzania	SAC	201	Kato-Katz, Direct, Mini-FLOTAC	None	Hookworm	1, 2
Brown et al. (2003)^b^	Uganda	all	412^a^	Kato-Katz, FEC	None	*A. lumbricoides, T. trichiura*, hookworm	1
Endris et al. (2013)	Ethiopia	SAC	354	Kato-Katz, Direct, FEC	Combined	*A. lumbricoides, T. trichiura,* hookworm	1
Funk et al. (2013)	India	all	110	Kato-Katz, FEC	None	*A. lumbricoides, T. trichiura*, hookworm	2
Habtamu et al. (2011)	Ethiopia	SAC	271	Kato-Katz, FLOTAC	Combined	*A. lumbricoides, T. trichiura*, hookworm	1
Jeandron et al. (2010)	Kyrgyzstan	SAC	71	3-sample Kato-Katz, FLOTAC	Combined	*A. lumbricoides*	1, 2
Knopp et al. (2008)	Tanzania (Zanzibar)	SAC	340	Kato-Katz, 2-sample Kato-Katz, 3-sample Kato-Katz, (Koga Agar)	Predicted estimate	*A. lumbricoides, T. trichiura*, hookworm	1
Knopp et al. (2009b)	Tanzania (Zanzibar)	SAC	279	3-sample Kato-Katz, FLOTAC	Combined	*A. lumbricoides, T. trichiura*, hookworm	1, 2
Knopp et al. (2011)	Tanzania (Zanzibar)	SAC	343, 269	2-slide Kato-Katz, FLOTAC	Combined	*A. lumbricoides, T. trichiura*, hookworm	1, 2
Knopp et al. (2014)	Tanzania	all	1179	2-slide Kato-Katz, FLOTAC, (PCR)	Combined, LCA	Hookworm	1
Levecke et al. (2011)	Brazil, Cameroon, Tanzania, Vietnam, India	SAC	350, 114, 199, 772, 101	Kato-Katz, McMaster	Combined	*A. lumbricoides, T. trichiura*, hookworm	1, 2
Machicado et al. (2012)	Peru	SAC	73	Kato-Katz, Direct, (SSTT)	Combined	*A. lumbricoides, T. trichiura*, hookworm	1
Neves Santos et al. (2005)	Brazil	SAC	258	3-slide Kato-Katz, Direct	Combined	*A. lumbricoides, T. trichiura*, hookworm	1
Pullan et al. (2010)^b^	Uganda	SAC,>20	853, 553^a^	Kato-Katz, 2-sample Kato-Katz	None	Hookworm	1,2
Utzinger et al. (2008)	Cote d’Ivoire	SAC	102	2-slide Kato-Katz, FEC, FLOTAC	Combined	Hookworm	1, 2
Von Schiller et al. (2013)	Colombia	SAC	90	Kato-Katz, FEC, Direct	Combined	*A. lumbricoides, T. trichiura*, hookworm	1

FEC, formol-ether concentration; SAC, school-aged children; SSTT, spontaneous sedimentation in tube technique.

a Split into several populations for analysis.

b Datasets contributed by authors.

### 3.2 LCA of diagnostic test sensitivities (presence of infection)

For all STH species, the models allowing for dependency between compared diagnostic tests showed a better fit, indicated by a lower DIC (not shown). Significant positive correlation between diagnostic test outcomes for infected individuals was observed, especially for comparisons of a 1-slide 1-sample Kato-Katz test with other diagnostic tests (details are provided in Supplementary Data S2).

**Fig. 3:**
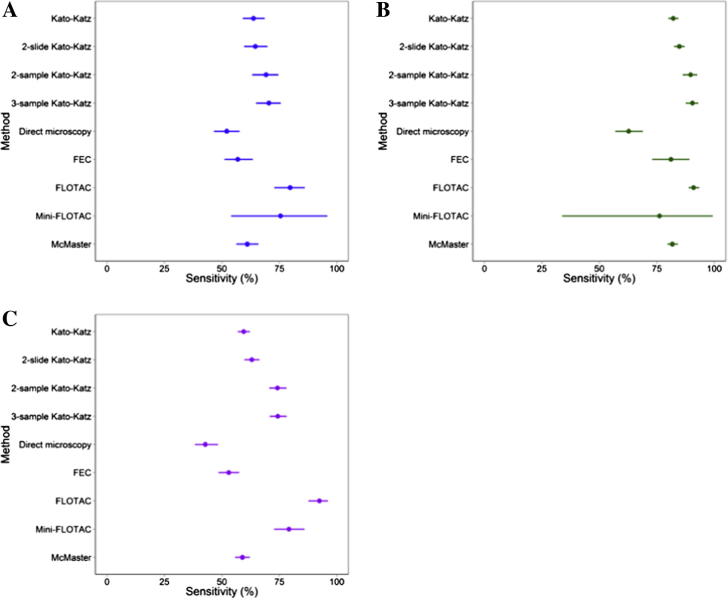
Sensitivity of selected diagnostic tests for the detection of *Ascaris lumbricoides* (A), *Trichuris trichiura* (B) and hookworm (C). The sensitivity estimates (%) and their 95% Bayesian credible intervals were obtained by Bayesian latent class analysis for each soil-transmitted helminth species.

Taking this dependency into account, the sensitivities of selected diagnostic methods were estimated separately for *A. lumbricoides*, *T. trichiura* and hookworm and are provided in Table 2 and Fig. 3. Generally, sensitivities of all compared tests were higher for *T. trichiuria* (Fig. 3B) than for hookworm (Fig. 3C) and *A. lumbricoides* (Fig. 3A). The obtained sensitivities were highest overall for the FLOTAC method with 79.7% (95% Bayesian credible interval (BCI): 72.8–86.0%), 91.0% (95% BCI: 88.8–93.5%), and 92.4% (95% BCI: 87.6–96.2%) for *A. lumbricoides*, *T. trichiura* and hookworm, respectively (Table 2). The lowest sensitivity was observed for the direct microscopy method with 52.1% (95% BCI: 46.6–57.7%), 62.8% (95% BCI: 56.9–68.9%), and 42.8% (95% BCI: 38.3–48.4%), respectively.

**Table 2:** Sensitivity estimates for selected diagnostic methods by helminth species. The sensitivity estimates and 95% Bayesian credible interval (BCI) were obtained for each soil-transmitted helminth species by Bayesian latent class analysis. Specificity was included as a fixed term based on model fit.

Number of comparisons	*Ascaris lumbricoides*		*Trichuris trichiura*		Hookworm	
	63		67		94	
Method	Sensitivity (%)	95%BCI	Sensitivity (%)	95%BCI	Sensitivity (%)	95%BCI
1-slide Kato-Katz	63.8	59.1–68.6	82.2	80.1–84.5	59.5	56.9–62.2
2-slide Kato-Katz	64.6	59.7–69.8	84.8	82.5–87.1	63.0	59.8–66.4
2-sample Kato-Katz	69.2	63.2–74.6	89.7	86.3–92.6	74.2	70.6–78.1
3-sample Kato-Katz	70.4	64.9–75.6	90.5	87.6–93.1	74.3	70.8–78.2
Direct microscopy	52.1	46.6–57.7	62.8	56.9–68.9	42.8	38.3–48.4
Formol-ether concentration (FEC)	56.9	51.1–63.5	81.2	73.0–89.2	53.0	48.6–57.5
FLOTAC	79.7	72.8–86.0	91.0	88.8–93.5	92.4	87.6–96.2
Mini-FLOTAC	75.5	54.0–95.9	76.2	33.9–99.4	79.2	72.7–85.9
McMaster	61.1	56.3–65.9	81.8	79.6–84.2	58.9	55.7–62.2
Specificity	99.6		97.5		98.0

The estimated sensitivity of the 2-slide 1-sample Kato-Katz test for *A. lumbricoides* was 64.6% (95% BCI: 59.7–69.8%), for *T. trichiura* was 84.8% (95% BCI: 82.5–87.1%) and for hookworm was 63.0% (95% BCI: 59.8–66.4%). These estimates were only a slight improvement upon the sensitivities of a 1-slide 1-sample Kato-Katz test. However, increased sensitivities could be observed for 1-slide Kato-Katz performed on two consecutive samples. The sensitivity for Kato-Katz tests performed on three consecutive samples was only slightly further improved.

Test specificities were not the main outcome and were fixed at 99.6% for *A. lumbricoides*, 97.5% for *T. trichiura* and 98.0% for hookworm, based upon model fit.

### 3.3 Effect of infection intensity on diagnostic test sensitivity

**Fig. 4:**
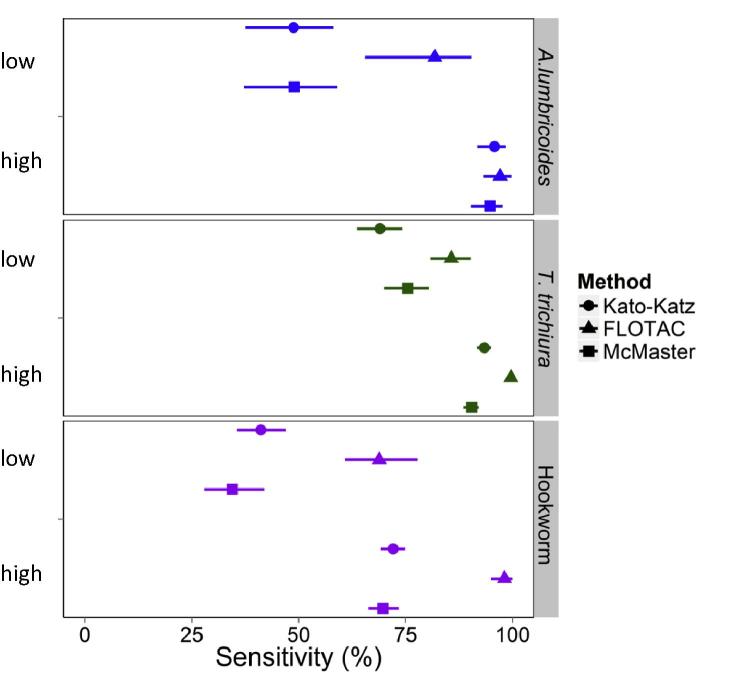
Sensitivity and 95% Bayesian credible intervals of a 1-slide 1-sample Kato-Katz, FLOTAC and McMaster test by intensity of infection and helminth species. The figure presents the results for those methods with sensitivity estimates in both intensity groups and for all three soil-transmitted helminth species. The Bayesian latent class analysis was performed, stratified by intensity of infection group, where high intensity was defined as ⩾2500 eggs per gram of faeces (epg), ⩾400 epg, and ⩾165 epg average infection intensity for *Ascaris lumbricoides*, *Trichuris trichiura* and hookworm, respectively.

The obtained sensitivity estimates by intensity group are presented in Table 3 and Fig. 4. For all tests and STH species evaluated in both intensity groups, sensitivity varied markedly and most strongly for the Kato-Katz method. For example, for *A. lumbricoides* the 1-slide Kato-Katz method had a sensitivity of 48.8% (95% BCI: 37.6–58.2%) in the low intensity group compared with 95.8% (95% BCI: 91.8–98.5%) in the high intensity group. Interestingly, in the low intensity group the sensitivity of Kato-Katz was improved markedly by performance of a second slide on the same sample. The sensitivity of the FLOTAC method was highest at 81.8% (95% BCI: 65.5–90.3%) at low intensity compared with 97.1% (95% BCI: 93.1–99.7%) at high intensity.

**Table 3:** Sensitivity estimates (Sens) by intensity of infection and helminth species. The sensitivity estimates and 95% Bayesian credible intervals (BCIs) were obtained for each soil-transmitted helminth species by Bayesian latent class analysis (LCA) stratified by intensity of infection group. High intensity was defined as ⩾2500 eggs per gram of faeces (epg), ⩾400 epg, and ⩾165 epg average infection intensity for *Ascaris lumbricoides*, *Trichuris trichiura* and hookworm, respectively. Specificity was included as a fixed term based on model fit. *n* indicates the number of comparisons for each of the methods.

Intensity group	*A. lumbricoides*						*T. trichiura*						Hookworm					
	Low			High			Low			High			Low			High		
Method	Sens (%)	95%BCI	*n*	Sens (%)	95%BCI	*n*	Sens (%)	95%BCI	*n*	Sens (%)	95%BCI	*n*	Sens (%)	95%BCI	*n*	Sens (%)	95%BCI	*n*
1-slide Kato-Katz	48.8	37.6–58.2	10	95.8	91.8–98.5	9	69.0	63.7–74.2	11	93.4	91.7–95.0	13	41.2	35.6–47.1	21	72.1	69.2–74.9	15
2-slide Kato-Katz	55.2	43.1–65.2	20	97.0	93.0–99.7	4	79.8	74.5–84.8	13	95.3	93.3–97.2	10	52.6	46.3–60.3	31	74.0	70.6–77.5	5
2-sample Kato-Katz	53.9	41.5–64.2	0^a^				71.3	65.9–77.0					55.8	48.2–63.4	3	88.6	83.0–92.6	5
3-sample Kato-Katz	58.4	47.3–67.7	2				73.5	68.2–78.9	1				56.1	48.4–63.7	1			
Direct microscopy	39.2	12.3–79.5	1				14.9	0.5–48.6	1				16.3	4.4–34.8	1	53.7	47.6–59.7	2
FEC	51.3	21.4–91.2	5				21.5	10.6–32.9	14				38.9	33.5–44.8	18			
FLOTAC	81.8	65.5–90.3	15	97.1	93.1–99.7	6	85.7	80.9–90.2	6	99.6	98.7–100	15	68.8	60.9–77.8	17	98.1	95.0–100	7
Mini-FLOTAC	47.1	28.7–69.2	8				58.3	18.7–95.8	2				44.3	34.3–56.4	11	97.3	91.0–99.9	2
McMaster	48.9	37.2–58.9	9	94.7	90.2–97.7	11	75.5	70.0–80.4	4	90.4	88.5–92.1	18	34.5	27.9–42.0	13	69.7	66.3–73.3	10
Specificity		99.8			95.1			99.6			86.1			98.6			96.1	

FEC, formol-ether concentration.

a No direct comparison was included in the model but parameters were estimated based on 1- and 3-sample Kato-Katz comparisons.

### 3.4 Comparison of quantitative test performances

**Fig. 5:**
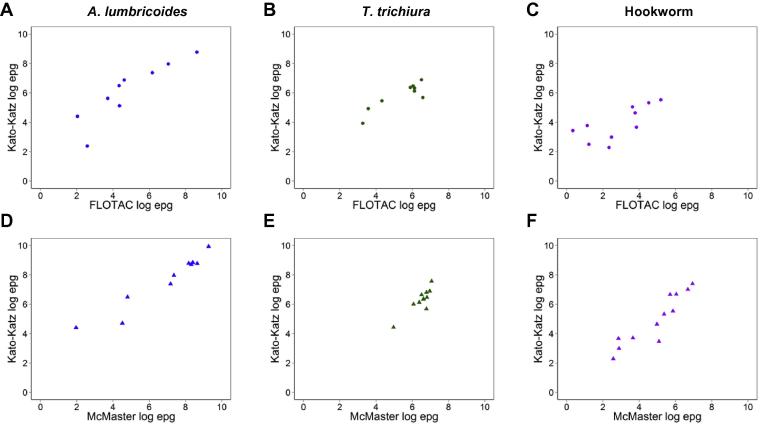
Scatter plots of log-transformed arithmetic mean egg counts (eggs per gram of faeces, epg) from studies comparing Kato-Katz with FLOTAC (A–C) and McMaster (D–F) tests. The graphs are presented separately for the three soil-transmitted helminth species, *Ascaris lumbricoides* (A, D), *Trichuris trichiura* (B, E) and hookworm (C, F). Arithmetic mean egg counts were log-transformed (natural logarithm) for presentation purposes. Due to the limited data analysed, we refrained from fitting a regression line and presenting linear regression coefficients.

A total of 17, 16 and 27 comparisons of average Kato-Katz *A. lumbricoides*, *T. trichiura* and hookworm egg counts with other diagnostic methods were obtained from 11 articles (Table 1, analysis 2). The majority of comparisons were between versions of Kato-Katz and FLOTAC or McMaster techniques. Only a few studies compared egg counts between Kato-Katz and FEC or Mini-FLOTAC methods; none with direct microscopy. Table 4 shows that the FLOTAC method generally underestimates the average egg counts compared with Kato-Katz, even though the difference is not statistically significant for *T. trichiura*. The McMaster technique, however, resulted in a higher egg count for six of 11 comparisons (55%) for *T. trichiura* and four of 12 comparisons (33%) for hookworm whilst *A. lumbricoides* egg counts were significantly lower. The relationships between the logarithmic average measurements of Kato-Katz and FLOTAC or McMaster techniques followed a linear trend as shown by the scatter plots presented in Fig. 5.

**Table 4:** Comparison of arithmetic mean egg counts (eggs per gram of faeces, epg) obtained by various techniques and Kato-Katz (various protocols). The statistical significance of the difference between egg counts was assessed using a Wilcoxon matched pairs signed-ranks test.

Method	*n*	Mean epg (range)	Mean Kato-Katz epg (range)	*n* lower (%)^a^	*n* higher (%)^a^	Difference *P* value
*Ascaris lumbricoides*						
FLOTAC	9	839 (8–5594)	1457 (11–6459)	8 (89)	1 (11)	0.011
McMaster	11	3456 (7–10643)	6990 (82–25079)	11 (100)	0	0.003
FEC	1	1	0	0 (0)	1 (100)	–
Mini-FLOTAC	1	47	80	1 (100)	0	–

*Trichuris trichiura*						
FLOTAC	9	359 (26–724)	439 (51–985)	7 (78)	1 (11)	0.139
McMaster	11	746 (143–1168)	693 (84–1938)	2 (18)	6 (55)	0.182
FEC	1	0.5	7	1 (100)	0	–
Mini-FLOTAC	1	0.3	0	0	1 (100)	–

*Hookworm*						
FLOTAC	10	43 (1–179)	87 (10–252)	8 (80)	1 (10)	0.013
McMaster	12	292 (13–1031)	418 (10–1630)	6 (50)	4 (33)	0.388
FEC	2	10 (6–14)	109 (62–156)	2 (100)	0	–
Mini-FLOTAC	2	220 (16–424)	241 (27–455)	1 (50)	0	–

*n*, number of comparisons; FEC, formol-ether concentration.

a To account for small fluctuations in egg counts, counts were considered as lower/higher than the Kato-Katz method if they were lower/higher than the Kato-Katz epg +/− 10%.

## 4 Discussion

A global assessment of STH diagnostic test sensitivities and their extent of variation is required to investigate the suitability of diagnostic tools for different transmission settings or stages of STH control programmes. Here we present, to our knowledge, the first meta-analysis of STH diagnostic method performance using a Bayesian LCA framework to overcome the absence of a true gold standard (Dendukuri and Joseph, 2001, Branscum et al., 2005). Our results demonstrate that sensitivities of evaluated diagnostic tests are low overall and cannot be generalised over different transmission settings. Sensitivity, overall and in both intensity groups, was highest for the FLOTAC method, but was comparable for Mini-FLOTAC and Kato-Katz methods. Test sensitivities are strongly influenced by intensity of infection and this variation needs to be taken into account for the choice of a diagnostic test in a specific setting. Moreover, reduced test sensitivity at low infection intensities is of increasing importance as ongoing control programmes reduce the prevalence and intensity of STH infections within endemic communities.

The Kato-Katz method is the most widely used and reported diagnostic method, due to its simplicity and low cost (Katz et al., 1972), and is recommended by the WHO for the quantification of STH eggs in the human stool (WHO, 2002). Even though the overall sensitivity of the Kato-Katz method was low, the results of the stratified analysis suggest a high sensitivity of 74–95% when infection intensity is high, which is likely the case for mapping and baseline assessment. However, the test sensitivity dropped dramatically in low transmission settings, making the method a less valuable option in later stages of control programmes. This is likely a reflection of methodological problems specific to the Kato-Katz method, especially when diagnosing multiple STH species infections, as different helminth eggs have different clearing times (Bergquist et al., 2009). In high intensity settings, little value was added by performing a 2-slide test on the same sample, even though this is the currently recommended protocol; whereas in low intensity settings sensitivity was improved by performing a second slide. Sensitivity increased significantly when performing the Kato-Katz method on multiple consecutive samples, which is most likely explained by daily variations of egg excretions and the non-equal distribution of eggs in the faeces leading to substantial variation in egg numbers between stool samples from the same person (Booth et al., 2003, Krauth et al., 2012).

For all investigated STH species, sensitivity was highest for the FLOTAC method, even when evaluated in low intensity settings, a finding which is consistent with previous evaluations (Utzinger et al., 2008, Knopp et al., 2009b, Glinz et al., 2010). However, despite its improved performance compared with other copro-microscopic methods, FLOTAC has several practical constraints including higher associated costs, necessity of a centrifuge and longer sample preparation time, decreasing its value as a universal diagnostic method (Knopp et al., 2009a). To enable its use in settings with limited facilities, the Mini-FLOTAC method, a simplified form of FLOTAC, was developed (Barda et al., 2013a). Our findings suggest that the sensitivity of Mini-FLOTAC is much lower than FLOTAC, and it does not outperform the less expensive Kato-Katz method according to a recent study in Kenya (Speich et al., 2010, Assefa et al., 2014). A recognised advantage of the Mini-FLOTAC method, however, is that it can be performed on fixed stools, enabling processing at a later date in a central laboratory. This can help to increase the quality control process and overcomes some of the logistical difficulties in examining fresh stool samples in the field on the day of collection (Barda et al., 2013a). The obtained Mini-FLOTAC sensitivity estimates have relatively high uncertainty, visible in the wide confidence intervals, probably due to the limited number of studies available for the analysis and their evaluation primarily in low transmission settings, where the number of positive individuals is very limited. The detection or failure of detection of a single individual therefore might have a large impact on the sensitivity estimate.

In remote areas where microscopy is often unavailable, studies can also use FEC, which allows the fixation of stool samples for later examination (WHO, 1994); several authors have also suggested the use of the McMaster technique as it is easier to standardise than Kato-Katz (Levecke et al., 2011, Albonico et al., 2012). Overall, the observed relative performances of these diagnostic tests when compared with the Kato-Katz method are consistent with those presented in the literature: the performance of Kato-Katz and McMaster methods were comparable, although this did vary by setting (Levecke et al., 2011, Albonico et al., 2013). Similarly, even though FEC had predominantly lower sensitivity than Kato-Katz in included studies, the reported relative performance varies in the literature (Glinz et al., 2010, Speich et al., 2013). The sensitivity of direct microscopy was consistently lower than the Kato-Katz method. Other available methods which were not included in our meta-analysis due to limited data availability, such as the midi-Parasep, do not show any improved test performance in their previous evaluations (Funk et al., 2013).

Although we present an improved approach for evaluating diagnostic test performances, accounting for the absence of a perfect gold standard by estimating the true unmeasured infection status and allowing for conditional dependency between the test outcomes, our analysis is subject to several limitations. The results presented here are limited by the low availability of comparable data for each diagnostic test, especially when performing the analysis stratified by intensity group. Direct microscopy was primarily evaluated in low intensity settings, which could have led to the lower observed sensitivity estimates, whereas the Kato-Katz method was evaluated in a full range of settings. The cut-off value to define high and low intensity groups of study populations was chosen based on the data included in the meta-analysis, but does not necessarily represent two main types of transmission settings. Nevertheless, the groupings demonstrate the substantial differences in test performance across varying infection intensities. As the investigated range of transmission settings was limited, further diagnostic test evaluations in specified transmission settings will be needed to provide concrete test performance estimates for each of the settings. To take into account the conditional dependency between compared diagnostic tests, we used a fixed effects model, assuming that conditional dependency is the same for all study settings. Different approaches allowing for varying correlations by using random effects to model sensitivities and specificities as a function of a latent subject-specific random variable could be explored further (Dendukuri and Joseph, 2001). Moreover, our findings might be biased towards results from studies comparing multiple diagnostic tests at the same time, as these are underpinned by a larger amount of data. Assumptions had to ensure identifiability of the model by limiting the number of parameters to be estimated. We focussed our analysis on the sensitivity of diagnostic tests, assuming that specificity of various methods do not differ largely, and therefore included the specificity of all single sample diagnostic tests as one fixed parameter. This assumption can be questioned, as for example Kato-Katz slides are more difficult to read than FLOTAC slides due to debris (Glinz et al., 2010); however, it is still an improvement on the assumption of 100% test specificity for all diagnostic tests as applied in previous publications (Booth et al., 2003, Knopp et al., 2011, Levecke et al., 2011). Using uninformative priors instead of fixed terms did not improve model fit and led to slightly wider BCIs.

Importantly, the current model assumes that sensitivities are identical within all populations, which is not fulfilled if sensitivity varies by study setting (Toft et al., 2005). Indeed, the stratified analysis showed that sensitivity varied by infection intensity; however, there were not sufficient data to obtain good estimates for all tests in various transmission settings. Additionally, sensitivity in a specific study setting might be affected by other factors including stool consistency and diet, standardisation and adherence to protocols, equipment quality and human error (Bogoch et al., 2006, Bergquist et al., 2009, Levecke et al., 2011). To overcome the limited comparability of evaluations from different studies, purposeful evaluations of test sensitivity over a continuous range of infection intensities in comparable populations, for example before and after treatment rounds, are clearly necessary to better refine sensitivity estimates, and could be used to identify intensity categories within which sensitivity remains comparable. Results could then be transformed into recommendations for the use of diagnostic tests for different stages of disease control programmes.

The performance of a diagnostic tool should not only be measured in terms of sensitivity, but also needs to consider the ability of the test to quantify faecal egg counts. Current infection and treatment effect indicators are based on the Kato-Katz method, and the question arises whether the increasing use of other methods will constitute a problem for standardised recommendations (WHO, 2002). The comparison of average egg counts obtained by Kato-Katz and FLOTAC methods shows a broad agreement with previous studies with generally higher Kato-Katz egg counts (Knopp et al., 2009b, Knopp et al., 2011, Albonico et al., 2013). The quantitative performance of the McMaster technique, however, varied in comparison to the Kato-Katz method as higher McMaster average egg counts were observed in several studies, especially for *T. trichiura* and hookworms (Levecke et al., 2011, Albonico et al., 2012, Albonico et al., 2013).

The current analysis has focussed on copro-microscopic diagnostic tests, which are based on examination of stool samples. There is current interest in developing more sensitive assays that allow a high sample throughput for screening of large populations using other biological samples and the simultaneous detection of several parasite species in co-endemic settings (Bergquist et al., 2009, Knopp et al., 2014). Recently, assays based on PCR have been developed for the detection of STH (Verweij et al., 2007, Schar et al., 2013, Knopp et al., 2014); however, we did not include this method in our meta-analysis due to limited data availability from field settings. Nonetheless, a recent study showed that the sensitivity of PCR methods was comparable with the Kato-Katz method, especially in low endemicity settings (Knopp et al., 2014).

In conclusion, we provide a first known meta-analysis of the sensitivity and quantitative performance of STH diagnostic methods most widely used in resource-limited settings. Our results show that the FLOTAC method had the highest sensitivity both overall and in low intensity settings; however this technique requires a centrifuge and has relatively low throughput. Our results further show that the sensitivities of the Kato-Katz and Mini-FLOTAC techniques were comparable and in high intensity settings both techniques provide a practical and reliable diagnostic method. A particular advantage of the Kato-Katz method is the ability to simultaneously detect STH and schistosome species at low cost; whereas the Mini-FLOTAC method has the advantage that it can be used on preserved samples. As control programmes reduce the intensity of infection, there is a need for diagnostic methods which are more sensitive than these currently used. In evaluating the performance of new diagnostic methods we recommend a standardised evaluation in multiple transmission settings, using the robust statistical methods presented here, as well as a consideration of the cost-effectiveness of alternative methods (Assefa et al., 2014).
