# Supplementary material for: Sensitivity of diagnostic tests for human soil-transmitted helminth infections: a meta-analysis in the absence of a true gold standard
Source: Int J Parasitol. 2014 Oct 1;44(11):765–74. doi: 10.1016/j.ijpara.2014.05.009 (PMC4186778; doi:10.1016/j.ijpara.2014.05.009)
Supplement: Supplementary Data S1 [file mmc1.docx]

**Supplementary Data S1.**

**1. Medical subject headings and search terms used to identify publications presenting the evaluation of diagnostic techniques for the human soil-transmitted helminths.**

*1.1. OvidSP (http://ovidsp.uk.ovid.com)*

(limit human)

exp Helminthiasis/ OR exp Helminths/ OR exp Ascariasis/ OR exp Trichuriasis/ OR exp Ascaris lumbricoides/ OR hookworm.tw. OR ascariasis.tw. OR trichuriasis.tw. OR Necator americanus.tw. OR Ancylostoma duodenale.tw. OR Ascaris lumbricoides.tw. OR Trichuris trichiura.tw. OR intestinal parasites.tw. OR geohelminths.tw. OR soil#transmitted helminth*

AND

exp "Diagnostic Techniques and Procedures"/ OR exp Parasite Egg Count/ OR diagnostic*.tw. OR Kato*.tw. OR Koga.tw. OR Concentration.tw. OR Flotac OR Microscopy OR Wet* OR direct

AND

exp "Sensitivity and Specificity"/ OR diagnosti*.tw. OR evaluation.tw. OR accuracy.tw. OR performance.tw. OR compari*.tw. OR sensitiv*.tw. OR specific*.tw.

*1.2. PUBMED (http://www.ncbi.nlm.nih.gov/pubmed)*

Helminthiasis[MH] OR Helminths[MH] OR Ascariasis[MH] OR Trichuriasis[MH] OR Ascaris lumbricoides[MH] OR hookworm[tw] OR ascariasis[tw] OR trichuriasis[tw] OR Necator americanus[tw] OR Ancylostoma duodenale[tw]. OR Ascaris lumbricoides[tw] OR Trichuris trichiura[tw] OR intestinal parasites[tw] OR geohelminths[tw] OR soil-transmitted helminth*[tw]

AND

Diagnostic Techniques and Procedures [MH] OR Parasite Egg Count[MH] OR diagnostic*[tw] OR Kato*[tw] OR Koga[tw] OR Concentration[tw] OR Flotac[tw] OR Microscopy[tw] OR Wet*[tw] OR direct[tw]

AND

Sensitivity and Specificity[MH] OR diagnosti*[tw] OR evaluation[tw] OR accuracy[tw] OR performance[tw] OR compari*[tw] OR sensitiv*[tw] OR specific*[tw]
