# Supplementary material for: Sensitivity of diagnostic tests for human soil-transmitted helminth infections: a meta-analysis in the absence of a true gold standard
Source: Int J Parasitol. 2014 Oct 1;44(11):765–74. doi: 10.1016/j.ijpara.2014.05.009 (PMC4186778; doi:10.1016/j.ijpara.2014.05.009)
Supplement: Supplementary Data S2 [file mmc2.docx]

Supplementary Data S2.

**1. Bayesian Latent Class Analysis (LCA) - description of the model**

A Bayesian latent class model was used to estimate the sensitivities of different diagnostic tests in the absence of a true gold standard as previously described in [Assefa et al. (2014](#_ENREF_1)). The models were developed based on the two dependent tests - one population probability model described by [Dendukuri and Joseph (2001](#_ENREF_3)) and [Branscum et al. (2005](#_ENREF_2)), which incorporates covariance terms to account for the dependency between compared diagnostic tests.

For each comparison of two diagnostic test methods *ij* the results were cross-classified in 2x2 tables as follows:

|  |  | ***I*** | |
| --- | --- | --- | --- |
|  |  | *+* | - |
| ***J*** | + | X*_k++_* | X*_k+-_* |
|  | - | X*_k-+_* | X*_k--_* |

In the model, each study population *k* has its own unobserved infection prevalence (π*_k_*). Where more than two tests were performed in the same population, separate tables were drawn for each comparison; the prevalence (π*_k_*) was however modelled jointly. $T_{j}^{+}$and $T_{j}^{-}$denote positive and negative test results from test *j,* and $D^{+}$and $D^{-}$denote true numbers of infected and non-infected individuals. The sensitivity and specificity of test *j* is defined as $S_{j}$ and $C_{j}$ where $S_{j}=\Pr(T_{j}^{+}|D^{+})$ and $C_{j}=\Pr(T_{j}^{-}|D^{-})$. The model assumes that $S_{j}$ and $C_{j}$ do not vary between different study populations, which was later tested by performing a stratified analysis by intensity of infection groups.

The joint distribution of the results ($X_{k++},X_{k+-},X_{k-+},X_{k--}$) of the two tests is multinomial, ${\left( X_{k++},X_{k+-},X_{k-+},X_{k--} \right)\sim Multi(p}_{k++},p_{k+-},p_{k-+},p_{k--}, N_{k})$ with the multinomial probabilities calculated as follows:

$p_{k++}=P\left( T_{i}^{+}, T_{j}^{+}| kth population \right)={[S}_{i}S_{j}+{{covD}_{ij}}^{+}]\pi_{k}+[\left( 1-C_{i} \right)\left( 1-C_{j} \right)+{{covD}_{ij}}^{-}](1-\pi_{k})$

$$p_{k+-}=P\left( T_{i}^{+}, T_{j}^{-}| kth population \right)={[S}_{i}\left( S_{j}-1 \right)-{{covD}_{ij}}^{+}]\pi_{k}+[\left( 1-C_{i} \right)C_{j}-{{covD}_{ij}}^{-}](1-\pi_{k})$$

$$p_{k-+}=P\left( T_{i}^{-}, T_{j}^{+}| kth population \right)=\left[ \left( S_{i}-1 \right)S_{j}-{{covD}_{ij}}^{+} \right]\pi_{k}+{[C}_{i}\left( 1-C_{j} \right)-{{covD}_{ij}}^{-}](1-\pi_{k})$$

$$p_{k--}=P\left( T_{i}^{+}, T_{j}^{+}| kth population \right)=\left[ \left( S_{i}{-1)(S}_{j}-1 \right)+ {{covD}_{ij}}^{+} \right]\pi_{k}+{[C}_{i}C_{j}+{{covD}_{ij}}^{-}](1-\pi_{k})$$

The probability of observing a concordant result is increased by the covariance ${{covD}_{ij}}^{+}=S_{ij}-S_{i}S_{j}$where $S_{ij}=\Pr\left( T_{i}^{+}T_{j}^{+} | D^{+} \right)$ for infected individuals and by ${{covD}_{ij}}^{-}=C_{ij}- C_{i}C_{j}$ where $C_{ij}=\Pr\left( T_{i}^{-},T_{j}^{-} | D^{-} \right)$ for non-infected individuals. The probabilities of observing discordant results are reduced by the same amounts. The conditional correlations between two test outcomes for infected and non-infected individuals are calculated as $\rho_{D^{+}}=\frac{covD^{+}}{\sqrt{S_{i}\left( {1-S}_{i} \right)S_{j}(1-S_{j})}}$ and $\rho_{D^{-}}=\frac{covD^{-}}{\sqrt{C_{i}\left( {1-C}_{i} \right)C_{j}(1-C_{j})}}$ , respectively.

Prior information for all test sensitivities $S_{j}$ and the population prevalence $\pi_{k}$ was uninformative using a beta distribution with the shape parameters alpha and beta equal 1. For the covariance terms, a uniform prior distribution was assumed with limits as described in [Branscum et al. (2005](#_ENREF_2)) and [Dendukuri and Joseph (2001](#_ENREF_3)) to ensure that probabilities are confined to values between 0 and 1. The specificity was included into the models as a fixed term as explained below.

*1.1. Sensitivity of repeated Kato-Katz tests*

For the Kato-Katz method, variations in the number of measurements were found in the data. For example Kato-Katz could be distinguished into 1-, 2- and 3- slide tests performed on a single sample, and up to three tests performed on different samples. These variations were included in the model using the sensitivity of a 1-sample 1-slide Kato-Katz test and additional adjustment terms$adjS$. The sensitivity of a 2-slide Kato-Katz test S_2_ was modelled as ${{S_{2}=S}_{1}+ {adjS}_{2}}$, where $S_{1}$ is the sensitivity of a 1-slide test. The limits of ${adjS}_{2}$ were set such that $S_{2}$cannot be lower than $S_{1}$, however it cannot exceed 100%. The same adjustment term ${adjS}_{2}$was used for a 3-slide Kato-Katz test, as no improvement in sensitivity by a third slide has been observed previously. The sensitivity of a 2-sample 1-slide Kato-Katz test was modelled as $S_{3}=1-((1-S_{1})(1-S_{1}) +{adjS}_{3})$ where S_1_ is the sensitivity of a 1-slide Kato-Katz test and ${adjS}_{3}$ is the covariance for two consecutive Kato-Katz tests. The prior limits for ${adjS}_{3}$ were set such that $S_{3}$ cannot be smaller than $S_{1}$ and that $S_{3}$ cannot exceed the sensitivity of two consecutive tests, assuming no dependency $\left( 1-\left( 1-S_{1} \right)*\left( 1-S_{1} \right) \right)$ (this simply means that ${adjS}_{3}=0$). Similarly, the sensitivity of a 3-sample Kato-Katz test $S_{4}$ was modelled as$S_{4}=1-((1-S_{3})*(1-S_{1}) +{adjS}_{4}).$

*1.2. Specificity*

To limit the number of parameters in the model to be estimated, assumptions were made about the specificity of diagnostic tests. Specificity was included as a fixed term based on the lowest deviance information criterion (DIC) value and was assumed to be the same for all compared single sample methods $C_{j (1 sample)}=C1$. The covariance was assumed to be the same for all 1-sample test comparisons ${{covD}_{ij}}^{-}={covD11}^{-}$ (e.g. for Kato-Katz versus FLOTAC and Kato-Katz versus formol-ether concentration (FEC)), but varied when comparing a 1-sample test with a 2-sample test ${(covD12}^{-})$ or a 1-sample test with a 3-sample test ${(covD13}^{-})$ etc. The specificity of a 2-sample Kato-Katz test was modelled as $C2={C1}^{2}+ {adjC}_{2}$, where $C2$ cannot be larger than the specificity of a single test $C1$ or smaller than the specificity of two consecutive tests, assuming no dependency. Similarly, the specificity of a 3-sample Kato-Katz test was modelled as $C3=C2C1+ {adjC}_{3}$. The specificity of a 2- and 3-slide Kato-Katz test was modelled as the specificity of a 2- and 3-sample test, respectively, as misdiagnosis occurs at the microscopy stage and not the sample preparation stage.

The models were built separately for hookworm, *Ascaris lumbricoides* and *Trichuris trichiura,* and computed using WinBUGS software (Spiegelhalter, D., Thomas, A., Best, N., Gilks, W., 1996. BUGS: Bayesian Inference Using Gibbs Sampling. MRC Biostatistics Unit, Cambridge). Two chains were run simultaneously using differing initial values. After allowing 10,000 iterations for model convergence, parameters were estimated in further 10,000 iterations storing every 10^th^ estimate. Parameter summary statistics were calculated based on these 1,000 stored estimates.

*1.3. Observed correlation in test comparisons*

For all soil-transmitted helminth (STH) species, the models allowing for dependency between compared diagnostic tests showed a better fit, indicated by a lower DIC. Significant positive correlation between diagnostic test outcomes for infected individuals was observed for 30 of 54 test comparisons and especially for comparisons of a single Kato-Katz test with other methods where 16 of 20 test comparisons had correlations significantly larger than 0. The values of positive correlations ranged from 0.02 to 0.83 and were highest comparing single sample with multiple sample Kato-Katz tests.

**References**

Assefa, L.M., Crellen, T., Kepha, S., Kihara, J.H., Njenga, S., Pullan, R.L., Brooker, S.J. 2014. Diagnostic performance and cost-effectiveness of alternative methods for detection of soil-transmitted helminths in a post-treatment setting in western Kenya. PLoS Negl. Trop. Dis., 8, e2843.

Branscum, A.J., Gardner, I.A., Johnson, W.O., 2005. Estimation of diagnostic-test sensitivity and specificity through Bayesian modeling. Prevent. Vet. Med., 68, 145 - 163.

Dendukuri, N., Joseph, L., 2001. Bayesian approaches to modeling the conditional dependence between multiple diagnostic tests. Biometrics 57, 158 - 167.
